# Changes in Cytokines Concentration Following Long-Distance Running: A Systematic Review and Meta-Analysis

**DOI:** 10.3389/fphys.2022.838069

**Published:** 2022-02-17

**Authors:** Micael Deivison de Jesus Alves, Devisson dos Santos Silva, Erika Vitoria Moura Pereira, Danielle Dutra Pereira, Matheus Santos de Sousa Fernandes, Dayane Franciely Conceição Santos, Davi Pereira Monte Oliveira, Lucio Marques Vieira-Souza, Felipe J. Aidar, Raphael Fabricio de Souza

**Affiliations:** ^1^Department of Physical Education, Federal University of Sergipe (UFS), São Cristóvão, Brazil; ^2^Graduate Program in Physical Education, Postgraduate Program in Physical Education, Federal University of Sergipe (UFS), São Cristóvão, Brazil; ^3^Group of Studies and Research of Performance, Sport, Health and Paralympic Sports—GEPEPS, Federal University of Sergipe (UFS), São Cristovão, Brazil; ^4^Department of Physiology and Pharmacology, Biological Sciences Course, Federal University of Piauí, Teresina, Brazil; ^5^Graduate Program, Postgraduate Program in Neuropsychiatry and Behavioral Sciences, Federal University of Pernambuco (UFPE), Recife, Brazil; ^6^Department of Nutrition, Federal University of Sergipe (UFS), São Cristóvão, Brazil; ^7^Physical Education Course, State University of Minas Gerais-UEMG, Passos, Brazil

**Keywords:** marathon, aerobic, endurance, cytokine, myokine, adipokine

## Abstract

Long-distance running is an exhausting effort for the whole organism. Prolonged aerobic exercise induces changes in inflammatory markers. However, predicting muscle damage in response has limitations in terms of selecting biomarkers used to measure inflammatory status. The present study conducts a systematic review and meta-analysis of articles focusing in ultra-marathon, marathon, and half-marathon and levels of cytokines. The search was conducted in PubMed, Web of Science, and Scopus databases, resulting in the inclusion of 76 articles. IL-6 was highlighted, evaluated in 62 studies and show increase in the standard mean difference (SMD): half-marathon (SMD −1.36; IC 95%: −1.82, −0.89, Ch^2^:0.58; tau^2^:0.00; *p* < 0.0001), marathon (SMD −6.81; IC 95%: −9.26, −4.37; Ch^2^:481.37 tau^2^:11.88; *p* < 0.0001) and ultra-marathon (SMD −8.00 IC 95%: −10.47, −5.53; Ch^2^:328.40; tau^2^:14.19; *p* < 0.0001). In contrast meta-regression analysis did not show relationship to the running distance (p = 0.864). The meta-analysis evidenced increase in the concentration of IL-1ra (*p* < 0.0001), IL-1B (*p* < 0.0001), IL-8 (*p* < 0.0001), IL-10 (*p* < 0.0001) and TNF-α (*p* < 0.0001). Reduction in IL-2 (*p* < 0.0001) and INF-y (*p* < 0.03) and no change in the IL-4 (*p* < 0.56). The number of studies evaluating the effect of adipokines was limited, however Leptin and Resistin were recurrent. The effects of an acute bout of prolonged aerobic exercise will protect against chronic systemic inflammation. The time to return to baseline values showed a substantial and dose-dependent relationship with run volume. The concentration of IL-6 was robustly studied and the marathon running was the most explored. Network of endocrine interactions in which circulating factors, released in extreme exercises, interplay through inter-organ crosstalk and physiologic changes were expressed. The running volume variability was able to modulate compounds that play a fundamental role in the maintenance of homeostasis and cell signaling.

## Introduction

Regular physical activity has been described as positive on protein and enzyme concentration, increases in insulin sensitivity, skeletal muscle glucose uptake (Turcotte and Fisher, 2008) and also for promoting a generalized anti-inflammatory state (Gleeson et al., 2011). In contrast, some negative effects are evidenced after complex competitions, resulting from the high volume and repeated physical efforts as occurs in ultra-marathons (>6 h of duration or >50 km) (Knechtle and Nikolaidis, 2018). These specific runs require great resistance and muscle contraction, resulting in microtraumas to the connective tissue, bone, and skeletal muscle with exercise-induced muscle damage (EIMD) (Smith, 2000; Suzuki et al., 2003; Järvinen et al., 2013).

The EIMD is related to the inflammatory response, characterized by the body's defense against an aggressor agent, whose objective is to promote repair of the damaged tissue (Cerqueira et al., 2020). The magnitude of this process is regulated by pro (about 1.5−24 h after exercise) and anti-inflammatory factors (from 24 to 72 h after exercise) (Zaldivar et al., 2006; Allen et al., 2015; Cerqueira et al., 2020), with cytokines being responsible for coordination, amplification, regulation of the magnitude, duration, and effect of inflammatory events (Moldoveanu et al., 2001). Cytokines are molecules produced by cells of the immune system, active musculature, and other tissues such as adipose tissue (de Oliveira dos Santos et al., 2021). In addition to an important modulating activity of inflammation, they regulate the activation of energy pathways that support this process (Petersen and Pedersen, 2005).

Adipose and skeletal muscle and tissue are the main endocrine organs that produce adipokines and myokines. These biomarkers can be detrimental or beneficial in the body and crosstalk between different tissues (Leal et al., 2018) acting on the endocrine, paracrine and autocrine pathways (de Oliveira dos Santos et al., 2021). Knowledge about the loss of cytokine homeostasis brings to light a better understanding of the metabolic disorders resulting from long-term running, resulting to marked changes in the concentration profile, which can be the basis of many physiological and pathophysiological disorders (Knechtle and Nikolaidis, 2018), questioning the real health benefit. These questions are relevant not only in ultra-marathons, but also in less volumes (i.e., marathons and half-marathons) (Suzuki et al., 2003; Kaufmann et al., 2021; Tanner et al., 2021).

Under normal conditions cytokines lead to a systemic anti-inflammatory state (Gomarasca et al., 2020). Becoming potentially permissive for optimizing body energy expenditure (Pedersen, 2019; Das et al., 2020) and for the protection of diseases associated with inflammation, insulin resistance and hyperlipidemia. On the other hand, alarming results are shown when exercise is performed in large volumes, a dramatic increase in interleukin (IL)-6 concentration is observed (Margeli et al., 2005), a decrease in the concentration of myostatin mRNA in the skeletal muscle (Allen et al., 2011), consistent acute tissue inflammatory lesion (Margeli et al., 2005; Papassotiriou et al., 2008; Goussetis et al., 2009) and EIMD (Suzuki et al., 2003). That is, indicators of the potentially more injurious condition in prolonged exercise (Kerschan-Schindl et al., 2009).

Understanding that broadening the discussion of this information is of great importance for sport physiologists, coaches and athletes, which show an increase in the number of practitioners and popularity. In this systematic review followed by a meta-analysis, we sought to verify the current state of investigation in relation to the effects of long-term running on cytokine concentration.

## Methods

### Search Strategy

The systematic review report was carried out based on the “Preferred Reporting Items for Systematic Reviews and Meta-Analyses statement” (PRISMA) (Page et al., 2021). A research was performed in the PubMed, Web of Science and Scopus databases, from September 1 to 13, 2021, using the following boolean operators (AND/OR) and terms: “marathon” OR “aerobic” OR “endurance” AND “cytokine” OR “myokine” OR “adipokine”, as well as the use of synonyms and related search terms, with no start date limit and, considering as the final year, the year 2021. The strategy of seeking additional articles in the gray literature and in the references of the papers found were adopted, with the aim of expanding the results.

Search results loaded into the online bibliographic management software Rayyan^TM^. After excluding duplicates, all titles and abstracts were independently analyzed by two investigators to determine the study's eligibility for inclusion in the review, in case of divergence a third author was consulted to establish a consensus. After these initial steps, the full texts were evaluated and the name of the first author, year of publication, title, objective, running distance, sample, runner's level, age, type of collection and inflammatory markers (pre, immediately after, after 24, 48, and 72 h).

### Eligibility Criteria

#### Abstract Selection

During the process of reading titles and abstracts, the following inclusion criteria were adopted: (I) studies involving running with distances equal to or >21 km and (II) measurement of pre and post-running inflammatory biomarkers.

#### Full-Text Articles Selection

As a second selection step, the studies were excluded for the following reasons: (1) pre-running collections with periods >7 days, (2) post-running with only periods >24 h, (3) jobs that showed running on a treadmill, (4) ingestion of drug, supplement or performance-maximizing drink, (5) runners with some pathology and (6) another sporting activity added to running. All parameters were evaluated in blood, urine, nasal and sputum samples collected before and after running.

### Risk of Bias Assessment

The recommendations of the Cochrane risk of bias assessment tool were followed, adopting the risk of bias strategy (Table 1) by two authors independently, and a third author was consulted to define the differences (Higgins et al., 2011) and the Review Manager program (RevMan5.3), developed for Systematic Reviews, which is available for free download (https://training.cochrane.org/online-learning/core-software-cochrane-reviews/revman/revman-5-download).

**Table 1:** Risk of bias evaluation of included studies.

**Bias domain**	**Source of bias**	**Support judgment**
Selection bias	Random sequence generation	The method used to generate the allocation sequence had sufficient detail to allow an evaluation and produce comparable groups
	Allocation concealment	The method used to conceal the allocation sequence, or detailing the intervention allocations could have been predicted
Bias performance	Blindness of participants	There was blind trial for participants and researchers
Bias detection	Result evaluation blindness	The measures used for the evaluation of results were blind
Frequency of friction	Incomplete results data	The conclusion of the results presented exclusions of analyzes or any other friction
Report Bias	Selective reports	How the selective results report was examined and what was found
Another type of bias	Anything else, ideally pre-specified	Important concerns about bias not covered in the other domains in the tool

### Statistical Analysis

The results were expressed as the standard mean difference (SMD) of the 95% confidence intervals (CI) presented by the Forest plot graph. For the analysis, 2 conditions were used: pre and post running. The studies that evaluated IL-6 were combined for trials with parameters (half-marathon, marathon, and ultra-marathon). To identify if the variables ultra-marathon, marathon and half-marathon could be significantly associated with effect size differences, a meta-regression analysis was performed. For studies with more than one intervention group, we considered only the control or placebo group without drug administration or other interventions. We used Cochran's (Ch^2^) and tau-square test (tau^2^) to assess heterogeneity. The *I*^2^ statistic was used to assess inconsistency (the percentage of the total variation of heterogeneity) of the effects of exercise (Higgins et al., 2003). We assessed visually and objectively the propagation of the risk of bias the symmetry graphically using funnel plots. The threshold for statistical significance was set to p <0.05.

## Results

### Search Results

**Figure 1:**
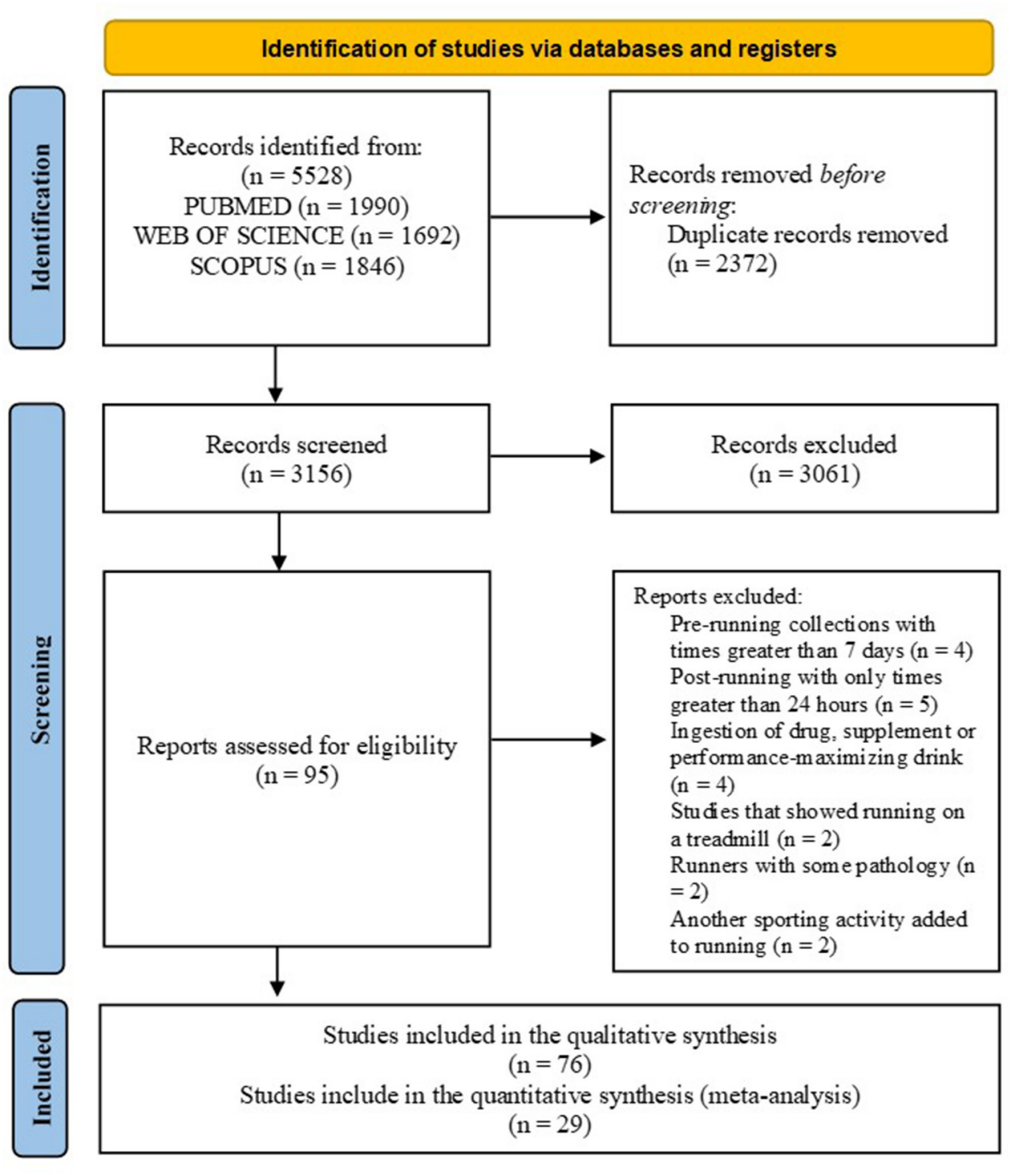
The search flowchart for screening process.

In the initial search, 5,528 articles were found, and 2,372 duplicate articles were excluded. Of 3,156 articles screened for eligibility, 3,061 were excluded based on title or abstract. The full texts of 95 potentially eligible studies were evaluated. Of these, 76 met the criteria and were included in the review, among which 29 made up the meta-analyses (Figure 1).

### Risk of Bias of Included Studies

**Figure 2:**
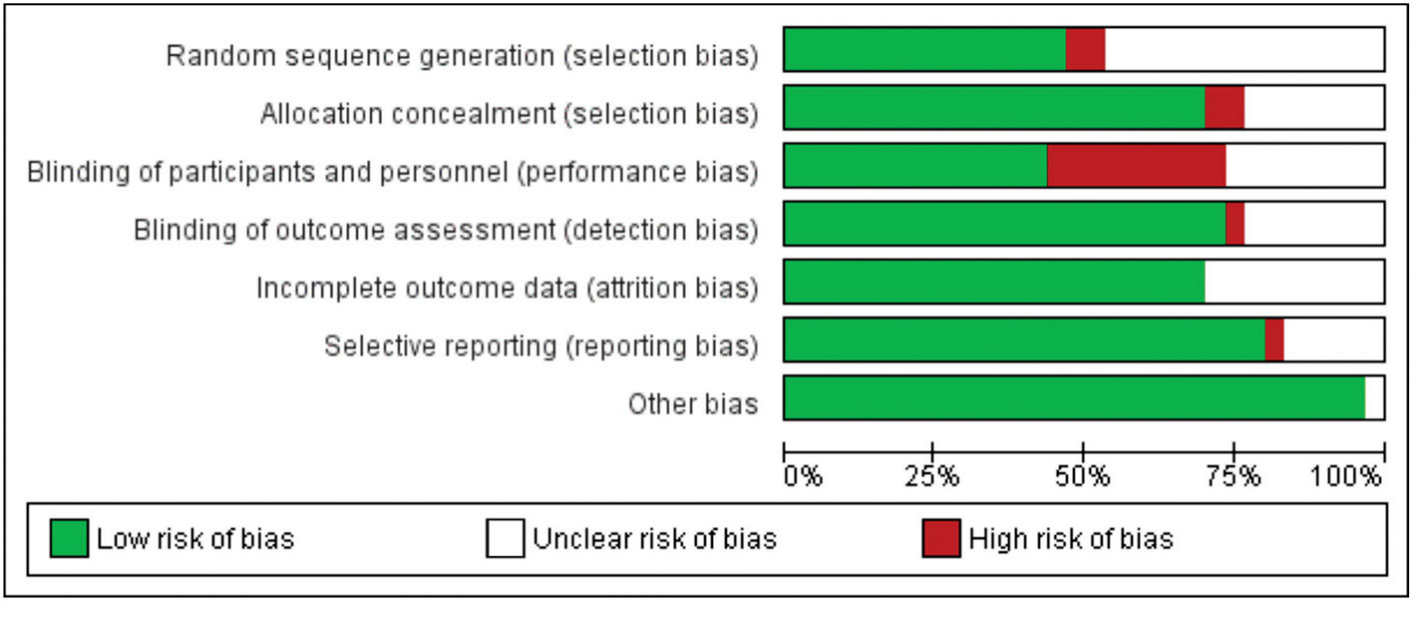
Risk of bias evaluation of included studies.

**Figure 3:**
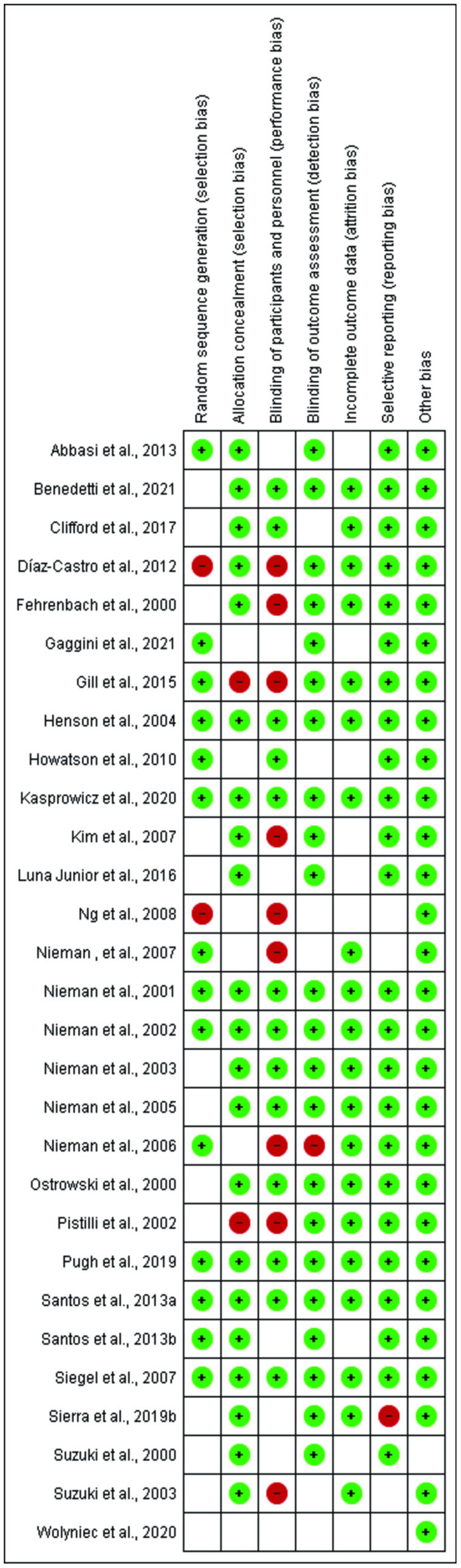
Risk of bias summary.

Of the 29 studies included, at least 10 studies were at risk of bias. Four studies had a high risk of random sequence generation and allocation concealment. Ten studies showed risk in blinding participants and researchers. When the blinding of results and selective notification was evaluated, two studies presented a risk. All studies had a low risk for incomplete outcome data. No study presented the risk of other biases (Figures 2, 3).

### Running Characteristic

Table 2 summarizes the results obtained from the 76 studies that investigated the relation to the degree of running volume in studies that evaluated cytokines in ultra-marathon, marathon, and half-marathon competitions. It was identified that the marathon had the highest proportion of articles investigated 39/76, followed by running of ultra-marathon 21/76 and half-marathon 9/76, four studies evaluated marathon and half-marathon (Bonsignore et al., 2002; Reihmane et al., 2013; Niemelä et al., 2016; Bekos et al., 2019), furthermore 2/76 evaluated the distance of 35 km (Miles et al., 2006; Yargic et al., 2019) and the study of Skinner et al. (2021) evaluated two runs 40 e 171 km.

**Table 2:** The study characteristics of included studies.

**Authors**	**Objective**	**Distance**	**No of participant**	**Age**	**Sample**	**Measure points^**^#^**^**	**Findings**
Santos et al. (2013b)	To investigate effects of DHA-Rich Fish Oil Supplementation on Lymphocyte Function Before and After a Marathon race.	Marathon	21	37 ± 2	Blood	3 to 7 days before and immediately after the running.	↓ IL-2, IL-10 and TNF-α
Bernecker et al. (2013)	To investigate blood parameters of healthy men before and immediately after a marathon race.	Marathon	15	43	Blood	Directly before and immediately after the running.	↑ IL-6 and TNF-α. ↔ Leptin
Nieman et al. (2006)	To measure the influence of ibuprofen use during the 160-km Western States Endurance Run on endotoxemia, inflammation, and plasma cytokines.	160 km	30	46 ± 2	Blood	The morning before the running and 10 to 15 min after the running.	↑ IL-6, IL-1ra, IL-8, IL-10, G-CSF, MCP-1 and MIP-1β. ↔ TNF-α
Shin and Lee (2013)	The aim of this study was to assess leukocyte chemotactic cytokine and leukocyte subset responses during ultra-marathon running.	308 km	60	52 ± 5	Blood	Before and immediately after the running.	↑ IL-6 and IL-10
Ng et al. (2008)	This study investigated changes in plasma LPS concentration and immune responses (leukocyte subsets and cytokines) during a half-marathon race in warm and humid conditions.	Half-marathon	32	25 ± 3	Blood	Before and immediately after the running.	↑ IL-6, IL-10 and IL-1ra; ↔ TNF-α and IL-1β
Nieman et al. (2007)	The purpose of this study was to measure the influence of quercetin on plasma cytokines, leukocyte cytokine mRNA, and related variables in ultramarathoners competing in the 160-km Western States Endurance Run.	160 km	63	44 ± 2	Blood	The morning before the test and 10 to 15 min after the running.	↑ IL-6, IL-1ra, IL-8, IL-10, TNF-α, G-CSF, MCP-1 and MIP-1β
Roupas et al. (2013)	To evaluate the effect of prolonged intensive aerobic exercise and acute energy deficit (ultra-marathon endurance race of 180 km distance) on serum leptin, adiponectin, resistin and visfatin levels.	180 km	17	51 ± 6	Blood	Running morning, post-running and 17 to 22 h after the running.	↑ Resistin; ↔ Adiponectin and Visfatin; ↓ Leptin
Niess et al. (1999)	We supposed that the down-regulation of the baseline concentration of HO-1 in athletes reflects an adaptional mechanism to regular exercise training.	Half-marathon	10	-	Blood	Before, immediately, 3 h and 24 h after the running.	↑ IL-8 immediately and then the values returned to baseline.
Sliwicka et al. (2021)	The aim of this study was to evaluate the effects of a marathon race on selected myokines and sclerostin in 10 male recreational runners.	Marathon	10	41 ± 7	Blood	24 h before, 24 and 72 h after the running.	↑ IL-6 and TNF-α
Ostrowski et al. (2000)	The present study included data from three marathon races to investigate the hypothesis that a relationship exists between running intensity and elevated concentrations of interleukin (IL)-6 in plasma.	Marathon	53	30	Blood	One week before, immediately, 1.5 h and 3 h after the running.	↑ IL-6 and IL-1rα at 1.5 h and then the values returned to baseline.
Zaccaria et al. (2002)	With the aim of clarifying the relationship between the level of EE and the reduction in leptin levels.	Half-marathon (HM) and 100 km	HM: 23 and 100 km: 11	HM: 44 ± 2; 100 km: 46 ± 3	Blood	Immediately before and immediately after the running.	HM: ↔ Leptin 100 km: ↓ Leptin
Czajkowska et al. (2020)	To evaluate the effect of continuous, prolonged, moderate-intensity running exercise, such as running a 100 km ultra-marathon, and acute energy deficit on serum levels of pro-inflammatory adipokines: resistin and chemerin.	100 km	15	42 ± 8	Blood	Before and after the running.	↑ Resistin; ↔ Chemerin
Vuolteenaho et al. (2014)	The aim of the present study was to investigate the effects of marathon running on the levels of adipokines adiponectin, leptin and resistin, as well as on markers associated with cartilage degradation in inflammatory arthritis and osteoarthritis.	Marathon	46	40 ± 9	Blood	1 day before and immediately after the running.	↑ Resistina and Adiponectin; ↔ Leptin
Vaisberg et al. (2013)	The aim of this study was to evaluate the immune response elicited by exhaustive exercise in different compartments, namely, the local (upper airway mucosa) and systemic (serum) compartments, by comparing athletes that presented or not with symptoms of upper airway disease after completing a marathon.	Marathon	22	41 ± 9	Blood (B) and nasal (N)	Before, immediately and 72 h after the running.	S: ↑ IL-6 and IL-10; N: ↑ IL-6; N: ↔ IL-10
Kim et al. (2007)	The present study evaluated muscle and cartilage biomarkers, and cytokine concentration during a 200 km running event.	200 km	54	45 ± 5	Blood	10–12 h before, immediately after the 100 km and at the end of the 200 km.	↑ IL-6; ↔ TNF-α
Starkie et al. (2001)	To investigate whether prolonged, strenuous running affects the ability of circulating monocytes to produce cytokines upon stimulation and whether spontaneous cytokine production is responsible, in part, for the increased plasma cytokine concentration.	Marathon	5	-	Blood	1 h before the running, immediately, 2 h and 24 h after the running.	↑ IL-6 at all periods after the running and TNF-α only at 2 h and then the values returned to baseline.
Gaggini et al. (2021)	Evaluate the changes in plasma levels of these bioactive lipids in healthy runners performing a half-marathon, at the end of the race and after 24 h recovery, and their associations with new recently proposed and common biomarkers of immune activation.	Half-marathon	13	47 ± 6	Blood	1 day before, immediately and 24 h after the running.	↑ IL-6 and fractalkine only immediately and then the values returned to baseline. ↔ TNF-α and VEGF-A
Castell et al. (1996)	The present study investigated white blood cell numbers, together with the plasma concentrations of some amino acids, cytokines and some acute phase response markers in athletes after two separate marathon races	Marathon	38	20-40	Blood	30 min before, 15 min, 1 h and 16 h after the running.	↑ IL-6 only immediately and 1 h and IL-2 only 16 h after the running. ↔ IL-2 only immediately and 1 h and IL-1α and TNF-α at all periods after the running.
Drenth et al. (1995)	Investigated whether a 6 h endurance race such as binding plasma cytokine changes and lipopolysaccharides (LPS) stimulated ex vivo cytokine production in a whole blood culture of 19 well-trained athletes.	51–86 km	71	43 ± 8	Blood	~18 h before and immediately after the running.	↑ IL-6 and IL-1ra; ↔ IL-1β and TNF-α
dos Santos et al. (2020)	To evaluate the prevalence of EIB in a group of recreational marathon runners without asthma, as well as to investigate both systemic and upper airway inflammatory responses and their correlation with marathon performance.	Marathon	38	38 [33-44]	Blood	24 h before and immediately after the running.	↑ IL-6, IL-8, IL-10 and TNF-α; ↔ IL-1β and IL-4
Uchakin et al. (2003)	To investigate the effects of marathon-associated stressors on cell-mediated versus humoral and anti-inflammatory versus pro-inflammatory balance, as well as their correlations with neuroendocrine response.	Marathon	15	39	Blood	24 h before, immediately, 1 h, 24 h, 48 h, 5 days and 8 days after the running.	↑ IL-6 only immediately and 1 h and TNF-α only immediately; ↔ IL-1β
Suzuki et al. (2003)	To investigate whether cytokines and neutrophils mediate exercise-related pathogenesis, we examined their responses and possible association after exhaustive exercise.	Marathon	10	31 ± 5	Blood (B) and urine (U)	1 day before and 10 min after the running.	B: ↑ IL-6, IL-8, IL-10, G-CSF, M-CSF and MCP-1; B: ↔ IL-1β, TNF-α; U: ↑ IL-6, IL-1β, IL-8, G-CSF, M-CSF and MCP-1; U: ↔ IL-10
Bonsignore et al. (2002)	Analyze whether the amount or duration of endurance exercise could modulate inflammatory and stress mediators, as well as circulating HPC counts.	Half-marathon (HM) and Marathon (Ma)	18	41 ± 13	Blood	9 ± 2 days before, immediately and ~24 h after.	HM: ↑ IL-6, TNF-α and G-CSF only immediately and then the values returned to baseline. Ma: ↑ IL-6 and G-CSF and then the values returned to baseline. Ma: ↔ TNF-α
Neidhart et al. (2000)	Compare cytokine response with cartilage oligomeric matrix levels protein (COMP) and melanoma inhibitory activity (MIA) after marathon.	Marathon	8	-	Blood	Before the running, after 31 km, after the running, 1 hour, 1 day and 2 days after the running.	↑ IL-6 only immediately; IL-1ra and TNF-α only immediately and 1 h, then the values returned to baseline. ↔ IL-1β, sTNFRII and sIL-6R
Fehrenbach et al. (2000)	To verify whether the regulation of basal HSP expression in immunocompetent cells exhibits adaptation due to regular endurance training.	Half-marathon	12	32 ± 9	Blood	24 h before, immediately, 3 h and 24 h after.	↑ IL-8 only immediately and then the values returned to baseline. ↔ TNF-α
Suzuki et al. (2000)	Investigate the mechanisms of exercise-induced immune perturbations.	Marathon	16	21–39	Blood	24 h before and after the running.	↑ IL-6, IL-1ra, IL-8, IL-10 and G-CSF; ↔ IL-1β, IL-2, IL-4 and IFN-γ
Sierra et al. (2019a)	Aimed at investigating whether marathon causes cardiac fatigue and, if it is the case, whether cardiac fatigue correlates with pulmonary levels of eNO and pulmonary inflammation.	Marathon	31	39 ± 9	Sputum	24 h before and immediately after the running.	↔ IL-6, IL-8; ↓ IL-12p40, IL-23 and IL-33
Clifford et al. (2017)	Examine whether beetroot juice (BTJ) would alleviate inflammation and muscle damage after a marathon.	Marathon	34	39 ± 12	Blood	Before, after, 24 and 48 h after the running.	↑ IL-6 only immediately and 24 h, IL-1β, IL-8 e IL-10, IFN-y and TNF-α only immediately, then the values returned to baseline. ↔ IL-1ra, IL-2, MCP-1 and IL-4
Santos et al. (2016)	To determine whether running a marathon race affects neutrophil function and to characterize the underlying mechanisms.	Marathon	23	34 ± 6	Blood	24 h before, immediately, 24 h and 72 h after the running.	↑ IL-6 and IL-8 only immediately and 24 h; IL-10 only immediately and then the values returned to baseline. ↔ IL-1β, IL-12 and TNF-α
Niemelä et al. (2016)	In order to shed more light on immune system function in response to acute exercise episodes, we compared pre- and post-race values of conventional and new biomarkers of immune activation, including suPAR, CD163, pro-inflammatory (IL−6, IL-8, tumor necrosis factor-α [TNF-α]), anti-inflammatory (IL-10, growth factor-β [TGF-β]), cytokines and muscle, cardiac, renal and hepatic status markers, among typical casual long-distance running event participants.	Half-marathon (HM) and Marathon (Ma)	HM: 4; Ma: 4	HM: 39 ± 13; Ma: 26 ± 15	Blood	24 h before, 3 h and 48 h after the running.	HM and Ma: ↑ IL-6, IL-8 and IL-10 only 3 h and then the values returned to baseline. ↔ TNF-α and TGF-β
Luna Junior et al. (2016)	Was to evaluate if there is some relation between RE and cytokine production in amateur marathon runners.	Marathon	22	34 ± 6	Blood	24 h before, immediately and 72 h after.	↔ IL-6, IL-1β, IL-4, IL-8, IL-10 and TNF-α at all periods after the running.
Shanely et al. (2014)	To measure the influence of RR supplementation on exercise-induced muscle damage, delayed onset of muscle pain (DMIT), plasma cytokines, and extracellular HSP72 (eHSP72) in experienced runners completing a marathon.	Marathon	48	43 ± 1	Blood	24 h before, immediately and 1,5 h after the Marathon.	↑ IL-8, MCP-1, IL-10, IL-6, G-CSF and eHSP72 at all periods after the running.
Abbasi et al. (2013)	Was studied the ability of blood cultures to produce cytokines in response to endotoxin (LPS) in athletes before, 30 min after, 3 h after and 24 h after a half-marathon.	Half-marathon	16 (8M; 8F)	34 ± 9 / 38 ± 5	Blood	Before, 30 min, 3 and 24 h after the running.	M: ↑ IL-6, MCP-1 and TGF-β only 30 min, IL-1ra and IL-8 only 30 min and 3 h and IL-10 at all periods after the running. ↔ IL-12p40, IL-12p70, TNF-α and IFN-y at all periods after the running. F: ↑ IL-6, IL-8 and MCP-1 only 30 min, IL-1ra only 3 h, IL-10 only 30 min and 3 h. F: ↔ IL-12p40, IL-12p70, TNF-α, IFN-y and TGF-β at all periods after the running.
Howatson et al. (2010)	The purpose of this study was to examine the effect of a tart cherry juice blend taken before and following running a Marathon on markers of muscle damage, inflammation, and oxidative stress.	Marathon	20	38 ± 5	Blood	24 h before, immediately, 24 and 48 h after the running.	↑ IL-6 only immediately and then the values returned to baseline.
Vaisberg et al. (2012)	Investigated the effects of acute exhaustive exercise on lipid transfer to HDL.	Marathon	14	38 ± 7	Urine	Before, immediately and 72 h after the running.	↑ IL-6 and TNF-α only immediately and then the values returned to baseline.
Costello et al. (2020)	Was to examine the effect of NZBC extract supplementation taken before and following running a half-marathon race on markers of EIMD.	Half-marathon	20	29 ± 7	Blood	Before, immediately, 24 and 48 h after the running.	↔ IL-6 at all periods after the running.
Cox et al. (2010)	To investigate the effectiveness of Difflam in alleviating post-race inflammatory responses and URS in trained runners competing in a half marathon.	Half-marathon	20	35 ± 8	Blood	24 h before and immediately after the running.	↑ IL-6, IL-1ra, IL-8 and IL-10
Peters et al. (2001)	To evaluate the effects of vitamin C supplementation on changes in circulating concentrations of cortisol, adrenaline, interleukin-10 (IL-10) and interleukin-1 receptor antagonist (IL-1Ra) that accompany the running in the ultramarathon were measured by immuno- chemiluminescence, radioimmunoassay and ELISA procedures.	90 km	29	39 ± 7	Blood	24 h before, immediately, 24 and 48 h after the running.	↑ IL-1ra and IL-10 only immediately and then the values returned to baseline.
Sierra et al. (2019b)	Was to determine the extent of association between the AGT Met235Thr, ACE I/D and BDKRB2 +9/−9 polymorphisms with inflammation, myocardial and muscle injury, induced by endurance exercise.	Marathon	81	39 ± 1	Blood	24 h before, immediately, 24, 72 h and 15 days after the running.	↑ IL-6, IL-1β, IL-8 and IL-10 only immediately and then the values returned to baseline. ↔ TNF-α and IL-12p70 at all periods after the running.
Wołyniec et al. (2020)	Investigate post-exercise proteinuria (PEP) after long exercise - marathon and ultramarathon races.	100 km	17	40 ± 4	Blood	Immediately before and after the running.	↑ IL-6
Pugh et al. (2019)	To evaluate the effects of probiotic supplementation on gastrointestinal (GI) symptoms, circulatory markers of GI permeability, damage and immune response markers during a marathon.	Marathon	24	36 ± 7	Blood	Before and immediately after the running.	↑ IL-6, IL-8 and IL-10
Scherr et al. (2011)	We investigated the kinetics of specific cardiac biomarkers (h-FABP, hs-cTnT, NT-proBNP), inflammatory markers (interleukin-10 (IL-10), IL-6, high-sensitive C-reactive protein (hs-CRP), and TNF-α), and a marker of renal dysfunction (cystatin C) before and up to 72 h after a marathon race in a large cohort of otherwise healthy individuals.	Marathon	102	42 ± 9	Blood	One week before, immediately, 24 and 72 h after.	↑ IL-6 and TNF-α only immediately and 24 h and IL-10 at all periods after the running.
Passos et al. (2019)	The present study was to investigate the association between quantity of macronutrient and micronutrient daily intake and inflammation induced by long-distance exercise.	Marathon	44	41 ± 1	Blood	24 h before, immediately, 24 and 48 h after the running.	↑ IL-6, IL-1β, IL-8 and IL-10 only immediately and then the values returned to baseline. ↔ IL-12p70 and TNF-α at all periods after the running.
Yargic et al. (2019)	This study is to determine serum levels of these molecules in runners after a long-distance trail run.	35 km	37	38 ± 10	Blood	24 h before and immediately after.	↑ IL-6, IL-15 and HSP72
Mastaloudis et al. (2004)	The present study was to determine whether exercise-induced lipid peroxidation and inflammation could be alleviated by 6 weeks of prior supple-mentation with vitamins E and C in recreationally trained women and men participating in an ultramarathon run.	50 km	22	39 ± 2	Blood	1 h before, immediately, 24, 48 and 72 h after the running.	↑ IL-6 only immediately and then the values returned to baseline.
Sansoni et al. (2017)	This study was to investigate and characterize the metabolic profile (in terms of hormones involved in energy metabolism), the metabolic inflammatory profile (in terms of adipokines), and the bone metabolism by comparing the OC-mediated response in experienced MUM runners, before and after a competition, with that of control subjects with a low PA profile.	65 km	17	38 ± 7	Blood	1 h before and immediately after the running.	↑ Visfatin and Resistin. ↓ Leptin
Miles et al. (2006)	This investigation was to determine whether attenuation of the IL-6 response to strenuous endurance exercise associated with exercise-induced muscle damage occurs in higher compared to lower *ad libitum* intake of carbohydrate.	35,2 km	32	Low CHO: 42 ± 15; high CHO: 33 ± 10	Blood	1 h before, immediately, 4 and 24 h after the running.	low CHO and high CHO: ↑ IL-6 only immediately and 24 h, then the values returned to baseline.
Santos et al. (2013a)	This study was to investigate the changes in lymphocyte and neutrophil selected functions before and after a marathon race.	Marathon	15	35 ± 3	Blood	3–7 days before and immediately after the running.	↑ IL-6 and IL-1ra; ↔ TNF-α
Gill et al. (2015b)	The study aimed to determine the circulatory endotoxin concentration and cytokine profile of ultra-endurance runners (UER, *n =* 19) and a control group (CON, *n =* 12) during a five stage 230 km ultra-marathon.	230 km	19	H: 41 ± 8; M: 49 ± 4	Blood	1 h before and immediately after the running.	↑ IL-6, IL-1ra, IL-1β, IL-10, TNF-α and IFN-γ
Díaz-Castro et al. (2012)	The present study was to determine for the first time and simultaneously whether oral CoQ10 supplementation may be efficient ameliorating the oxidative stress and pro-inflammatory effects induced by the strenuous exercise.	50 km	10	39 ± 2	Blood	Immediately before and immediately after the running.	↑ IL-6, IL-1ra and TNF-α
Nieman et al. (2001)	This study was to investigate the influence of carbohydrate, gender, and age on cytokine changes in a large group of runners after two competitive marathon races.	Marathon	50	42 ± 1	Blood	Immediately before and immediately and 1,5 h after the running.	↑ IL-6, IL-1ra, IL-1β, IL-10 and TNF-α at all periods after the running.
Skinner et al. (2021)	This study was to describe and compare the effects of a trail (40 km) race and an ultra-trail (171 km) race on leukocyte concentrations and cytokine profiles.	40 km and 171 km	40 km: 11 and 171 km: 12	40 km: 37 ± 9; 171 km: 38 ± 6	Blood	Immediately before and immediately after the running.	40 km: ↑ IL-6, IL-8 and TNF-α 40 km: ↔ IL-1β, MIP-1β, MCP-1, IL-2, IFN-γ, IL-4, IL-7, IL-17a, IL-3, IL-5, IL-12 and GM-CSF 171 km: ↑ IL-6, IL-1β, MCP-1, IL-8, MIP-1β, IL-4, IL-7, IL-17a and TNF-α; 171 km: ↔ IFN-γ, IL-3, IL-2, IL-5, IL-12 and GM-CSF
Larsen et al. (2020)	Acute and adaptive changes in systemic markers of oxidatively generated nucleic acid modifications [i.e., 8-oxo-7,8-dihydro-2'-deoxyguanosine (8-oxodG) and 8-oxo-7,8-dihydroguanosine (8-oxoGuo)] as well as inflammatory cytokines (i.e., C-reactive protein, interleukin-6, interleukin-10, and tumor necrosis factor alpha), a liver hormone [i.e., fibroblast growth factor 21 (FGF21)], and bone metabolism markers (sclerostin, osteocalcin, C-terminal telopeptide, and N-terminal propeptide of type 1 procollagen) were investigated following a marathon in 20 study participants.	Marathon	20	29 [24–37]	Blood	3–7 days before and immediately after the running.	↑ IL-6, IL-10 and TNF-α
Donnikov et al. (2009)	We studied changes in the levels of IL-6, LIF, and SCF during long exercise.	51–81 km	6		Blood	24 h before and immediately after the running.	↑ IL-6
Tavares-Silva et al. (2021)	Was to verify the effects of probiotic supplementation on cytokine production by monocytes and infections in the upper respiratory tract after an acute strenuous exercise.	Marathon	7	38 ± 3	Blood	24 h before, immediately and 1 h after the running.	↑ IL-10 only immediately and TNF-α at all periods after the running. ↔ IL-2 and IL-4 at all periods after the running.
Ostrowski et al. (1998)	Was performed to test the hypothesis that the cytokine response is locally produced in response to mechanically damaged myofibres or disrupted connective tissue in the muscle, and that a local cytokine response initiates the systemic inflammatory response.	Marathon	16	30 ± 1	Blood	1 week before, immediately and 2 h after the running.	↑ IL-6, IL-1ra and TNF-α at all periods after the running and IL-1β only immediately, then the values returned to baseline.
Bekos et al. (2019)	This study was to investigate the incidence of EIB in non-asthmatic non-professional runners and to study the association of EIB and changes in cytokine concentrations, skin or core temperature.	Half-marathon (HM) and Marathon (Ma)	HM: 36; Ma: 34	HM:36 ± 7; Ma:36 ± 7	Blood	24 h before and immediately after the running.	HM and Ma: ↑ HSP70 and HSP27
Siegel et al. (2007)	Exercise-associated hyponatremia (EAH), as defined by a blood sodium concentration [Na+] <135 mmol/L, may lead to hypotonic encephalopathy with fatal cerebral edema. Understanding the pathogenetic role of antidiuresis may lead to improved strategies for prevention and treatment.	Marathon	33	49 ± 10	Blood	24 h before, 2 and 24 h after the running.	↑ IL-6 only 2 h and then the values returned to baseline.
Nieman et al. (2003)	Changes in immune and oxidative stress parameters were measured in ultramarathon runners competing in the 160-km Western States Endurance Run.	160 km	45	46 ± 1	Blood	24 h before and immediately after the running.	↑ IL-6, IL-1ra, IL-8 and IL-10
Zimmer et al. (2016)	Investigates the short-term effects of a half marathon on immune cell proportions, pro-inflammatory cytokine levels, and recovery behavior of patients with breast cancer in the after race compared to healthy controls.	Half-marathon	9	47 ± 5	Blood	Immediately before, immediately and 24 h after the running.	↑ IL-6 only immediately and TNF-α only immediately and then the values decreased from the baseline.
Reihmane et al. (2013)	To test whether there were relations between endurance exercise-induced changes in the afore-mentioned mediators.	Half-marathon (HM) and Marathon (Ma)	HM: 22 and Ma: 18	HM: 26 ± 5; Ma: 27 ± 5	Blood	2 days before, immediately and 28 h after the running.	HM: ↔ IL-6 and TNF-α; Ma: ↑ IL-6 only immediately and then the values returned to baseline.
Wilhelm et al. (2014)	We measured SAPWD as a surrogate for atrial conduction and remodeling in healthy runners before and after a strenuous mountain marathon.	Marathon	10	34 ± 4	Blood	24 h before, immediately and 24 h after the running.	↑ IL-6 and TNF-α only immediately and then the values returned to baseline.
Chiu et al. (2013)	To measure the magnitude of serological response in ultra-mara-thon runners, compare the liver function tests, muscle damage markers and oxidative stress cytokines of athletes.	100 km	18	46 ± 9	Blood	1 week before, immediately and 24 h after the running.	↑ IL-6 and TNF-α all periods after the running.
Pistilli et al. (2002)	To examine the effects of a competitive marathon race on immune alterations in a relatively large group of younger and older runners.	Marathon	Older: 23 Young:75	Older: 57 Young: 37	Blood	Before, immediately and 1,5 h after the running.	Older and Young: ↑ IL-6, IL-1ra, IL-8 and IL-10 all periods after the running.
Henson et al. (2004)	To verify the influence of 6% carbohydrate intake and age on PHA-induced lymphocyte proliferation and cytokine production *in vitro*.	Marathon	25	41 ± 2	Blood	Before, immediately and 1,5 h after the running.	↓ IFN-γ all periods after the running.
Nieman et al. (2002)	To measure the influence of vitamin C compared with placebo supplementation on oxidative and immune changes in ultramarathoners competing in an ultramarathon race.	80 km	13	45 ± 2	Blood	Before and immediately after the running.	↑ IL-6, IL-1ra, IL-8 and IL-10; ↓ IL-2 and IFN-γ
Nickel et al. (2012)	To assess exercise-induced alterations of circulating dendritic cell (DC) sub-populations and toll-like receptor (TLR) expression after marathon running.	Marathon	16	E: 40 ± 7; NE: 40 ± 6	Blood	2–5 days before, immediately and 24 h after the running.	E and NE: ↑ IL-6 all periods after the running and IL-10 only immediately and TNF-α only 24 h.
Nieman et al. (2005)	Test these relationships, reasoning that elevations in plasma cytokines and significant muscle damage would occur within the first few hours of this high altitude race in the Sierra Nevada Mountains, and then be maintained for 20–30 h when correlations with CPK could be tested at the end of the race.	160 km	60	45 ± 1	Blood	The morning before the running and immediately after the running.	↑ IL-6, IL-1ra, IL-8, IL-10, MCP-1, MIP-1β and G-CSF
Toft et al. (2000)	Was to investigate whether fish oil supplementation was able to modulate the acute-phase response to strenuous exercise.	Marathon	10	28 [24–43]	Blood	One week before, immediately, 1.5, and 3 h after the running.	↑ IL-6, IL-1ra and TNF-α all periods after the running. ↔ TGFβ all periods after the running.
Bachi et al. (2015)	To investigate how physical and psychological changes induced in mara-thon runners by training and by the race can affect mood states, hormones and cytokines.	Marathon	20	35 ± 9	Blood	24 h before, immediately, and 72 h after the running.	↑ IL-10 only immediately and then the values returned to baseline. ↔ IL-8 only immediately and then the values decreased from the baseline.
Scherr et al. (2012)	To determine whether ingestion of NAB polyphenols for 3 weeks before and 2 weeks after a marathon would attenuate postrace inflammation and decrease URTI incidence.	Marathon	63	42 [35–49]	Blood	1 week before, immediately, 24 and 72 h after the running.	↑ IL-6 only immediately and then the values returned to baseline.
Ostrowski et al. (1999)	Investigates to what extent and by which time course prolonged strenuous exercise influences the plasma concentration of pro-inflammatory and inflammation responsive cytokines as well as cytokine inhibitors and anti-inflammatory cytokines.	Marathon	10	28 [24–37]	Blood	1 week before, immediately and every 30 min until 4 h after the running.	↑ IL-6 and IL-1ra all periods after the running, IL-1β only immediately and 30 min and TNF-α only immediately and until 3 h after the running, then the values returned to baseline.
Batatinha et al. (2020)	To evaluate the alterations caused by a marathon in the lymphocyte population and function, and the effects of probiotics in this process.	Marathon	13	40 ± 7	Blood	24 h before and 1 h after the running.	↑ IL-6, IL-8 and IL-10; ↔ IL-2, IL-4, IL-1β, TNF-α, IFN-γ and IL-15
Benedetti et al. (2021)	Monitored for the first time in ultramarathon athletes running the 24-h competition, an extremely demanding race in terms of muscular and physiological exertion.	99–218 km	22	42 ± 11	Blood	3 h before and immediately after the running.	↑ IL-6
Kasprowicz et al. (2020)	To examine whether two-week high-dose supplementation (10,000 IU/day) of vitaminD3can have an influence on 25 (OH)D serum concentration, and secondly, whether it can aecthepcidin, iron, and IL-6 responses to a 100-km ultra-marathon.	100 km	10	42 ± 8	Blood	Before, immediately and 12 h after the running.	↑ IL-6 only immediately and then the values returned to baseline.
Gill et al. (2015a)	To determine circulatory endotoxin concentration and cytokine profile of ultra-endurance runners in response to a 24-h continuous ultra-marathon competition conducted in temperate ambient conditions; and additionally, to determine the relationship between these responses with gastrointestinal symptoms.	122–208 km	17	40 ± 7	Blood	Before and immediately after the running.	↑ IL-6, IL-1ra, IL-1β, IL-8, IL-10 and TNF-α; ↔ IFN-γ

# *, Only until 72 hours after; ↑, Significant increase; ↓, Significant decrease; ↔, no change; IL, Interleukin; IL-1ra, Interleukin-1 receptor alfa; TNF-α, tumor necrosis factor- α; IFN-γ, interferon gamma; G-CSF, granulocyte colony-stimulating factor; MCP-1, monocyte chemotactic protein 1; MIP-1β, macrophage inflammatory protein 1 beta; TGF-β, transforming growth factor-β; VEGF-A, Vascular endothelial growth factor-A; sTNFRII, tumor necrosis factor type II p75; sIL-6R, interleukin-6 receptor gp80; HSP, heat shock protein; GM-CSF, granulocyte-macrophage colony-stimulating factor; B, blood; N, nasal; U, urine; HF, half-marathon; Ma, marathon; M, male; F, female; CHO, carbohydrate*.

From the articles that evaluated ultra-marathon running, four studies (Drenth et al., 1995; Donnikov et al., 2009; Gill et al., 2015a; Benedetti et al., 2021) evaluated distances in the ranges of 51–86, 51–81, 122–208, and 99–218 km respectively, two studies (Mastaloudis et al., 2004; Díaz-Castro et al., 2012) 50 km distance, one study (Sansoni et al., 2017) 65 km distance, one study (Nieman et al., 2002) 80 km distance, one study (Peters et al., 2001) 90 km distance, four studies (Chiu et al., 2013; Czajkowska et al., 2020; Kasprowicz et al., 2020; Wołyniec et al., 2020) evaluated 100 km, four studies (Nieman et al., 2003, 2005, 2006, 2007) evaluated 160 km, one study (Roupas et al., 2013) evaluated 180 km, one study (Kim et al., 2007) evaluated 200 km, one study (Gill et al., 2015b) evaluated 230 km and one study (Shin and Lee, 2013) evaluated 308 km. Some studies that evaluated the ultra-marathons presented particularities in relation to the topography characteristic. Four studies were carried out in mountains with uphill (5.500 meters) and dowhill (6.500 meters) (Nieman et al., 2003, 2005, 2006 and Nieman et al., 2007). Three studies reported parts with uphill by 2,800 meters (Díaz-Castro et al., 2012); 4,000 meters (Sansoni et al., 2017) and 10000 meters (Skinner et al., 2021). Four studies on trails with the varying ground (Mastaloudis et al., 2004; Roupas et al., 2013; Gill et al., 2015a,b). Five studies with flat ground, including athletics tracks (Chiu et al., 2013; Czajkowska et al., 2020; Kasprowicz et al., 2020; Wołyniec et al., 2020; Benedetti et al., 2021), the other studies did not identify the running ground.

### Runner's Level and Sex

Experienced runners, athletes, trained and well-trained were evaluated in 22 studies (Niess et al., 1999; Fehrenbach et al., 2000; Ostrowski et al., 2000; Suzuki et al., 2000, 2003; Toft et al., 2000; Bonsignore et al., 2002; Zaccaria et al., 2002; Nieman et al., 2003, 2005, 2006, 2007; Cox et al., 2010; Abbasi et al., 2013; Chiu et al., 2013; Roupas et al., 2013; Santos et al., 2013b; Shanely et al., 2014; Bachi et al., 2015; Sansoni et al., 2017; Passos et al., 2019; Gaggini et al., 2021). Amateur, recreational runners were present in 13 studies (Drenth et al., 1995; Castell et al., 1996; Starkie et al., 2001; Bonsignore et al., 2002; Kim et al., 2007; Díaz-Castro et al., 2012; Vaisberg et al., 2012, 2013; Reihmane et al., 2013; Vuolteenaho et al., 2014; Luna Junior et al., 2016; dos Santos et al., 2020; Sliwicka et al., 2021). Other characteristics such as experience ranging from 1 to 16 years, finalists, and time in a marathon running of <5 h were adopted in three studies respectively (Uchakin et al., 2003; Pugh et al., 2019; Wołyniec et al., 2020). The other studies did not presented the levels of the evaluated runners. Women were present in 35% of the studies. However, only the study by Abbasi et al. (2013) broken down the results by sex.

### Running Time

Mean running conclusion times were reported in 68% of the studies. In the half-marathon, the mean times varied between 1:30 and 2:12 h (Zaccaria et al., 2002; Ng et al., 2008; Cox et al., 2010; Abbasi et al., 2013; Reihmane et al., 2013; Niemelä et al., 2016; Costello et al., 2020; Gaggini et al., 2021). In marathons the mean times varied between 2:52 and 4:41 h (Castell et al., 1996; Toft et al., 2000; Pistilli et al., 2002; Henson et al., 2004; Howatson et al., 2010; Scherr et al., 2011, 2012; Nickel et al., 2012; Bernecker et al., 2013; Reihmane et al., 2013; Santos et al., 2013a,b; Vaisberg et al., 2013; Shanely et al., 2014; Vuolteenaho et al., 2014; Wilhelm et al., 2014; Niemelä et al., 2016; Clifford et al., 2017; Passos et al., 2019; Pugh et al., 2019; Sierra et al., 2019a; Batatinha et al., 2020; dos Santos et al., 2020; Larsen et al., 2020; Sliwicka et al., 2021; Tavares-Silva et al., 2021). In ultra-marathons, the mean times ranged between 6:00 and 62:20 h (Drenth et al., 1995; Peters et al., 2001; Nieman et al., 2002, 2003, 2005, 2006, 2007; Zaccaria et al., 2002; Mastaloudis et al., 2004; Kim et al., 2007; Donnikov et al., 2009; Chiu et al., 2013; Shin and Lee, 2013; Gill et al., 2015a; Czajkowska et al., 2020; Wołyniec et al., 2020; Benedetti et al., 2021; Skinner et al., 2021), with distances of 51–308 km. Three studies evaluated the specific distances of 35, 35.2 and 40 km, with the following mean conclusion times 5:08 (Yargic et al., 2019), 6:10 (Miles et al., 2006) and 6:50 hours (Skinner et al., 2021) respectively.

### Effects of Long-Distance Running on Cytokine Concentration

The effects of long-distance running on cytokine concentration were evaluated in 76 studies. The analyzed results comprised a period of up to 72 h after the running. The concentration of 35 cytokines was identified (IL-6, IL-8, IL-1β, IL-1ra, IL-2, IL-4, IL-10, IL-12, IL-12p40, IL-12p70, IL-23, IL-33, IL-15, IL-7, IL-17a, IL-3, IL-5, Tumor Necrosis Factor-Alfa (TNF-α), Interferon Gamma (IFN-γ), Granulocyte Colony-Stimulating Factor (VEGF-A), Fractalkine, Leptin, Resistin, Adiponectin, Visfatin, Tumor Necrosis Factor Type II p75 (sTNFRII), Interleukin-6 Receptor gp80 (sIL-6R), Transforming Growth Factor-Beta (TGF-β), Granulocyte-Macrophage Colony-Stimulating Factor (GM-CSF), Granulocyte Colony-Stimulating Factor (G-CSF), Monocyte Chemotactic Protein 1 (MCP-1), Macrophage Inflammatory Protein 1 Beta (MIP-1β), Heat Shock Protein (HSP)-72, HSP27 and HPS70). The review shows that long-term runs can change the volume of cytokine concentration, presenting as relevant factors the distance of the run and the recovery time until the analysis (Table 2).

### Interleukin 6

The most evaluated cytokine was IL-6 found in 62 studies (Drenth et al., 1995; Castell et al., 1996; Ostrowski et al., 1998, 1999, 2000; Neidhart et al., 2000; Suzuki et al., 2000, 2003; Toft et al., 2000; Nieman et al., 2001, 2002, 2003, 2005, 2006, 2007; Starkie et al., 2001; Bonsignore et al., 2002; Pistilli et al., 2002; Uchakin et al., 2003; Mastaloudis et al., 2004; Miles et al., 2006; Kim et al., 2007; Siegel et al., 2007; Ng et al., 2008; Donnikov et al., 2009; Cox et al., 2010; Howatson et al., 2010; Scherr et al., 2011, 2012; Díaz-Castro et al., 2012; Nickel et al., 2012; Vaisberg et al., 2012, 2013; Abbasi et al., 2013; Bernecker et al., 2013; Chiu et al., 2013; Reihmane et al., 2013; Santos et al., 2013a, 2016; Shin and Lee, 2013; Wilhelm et al., 2014; Gill et al., 2015a,b; Luna Junior et al., 2016; Niemelä et al., 2016; Zimmer et al., 2016; Clifford et al., 2017; Passos et al., 2019; Pugh et al., 2019; Sierra et al., 2019a,b; Yargic et al., 2019; Batatinha et al., 2020; Costello et al., 2020; dos Santos et al., 2020; Kasprowicz et al., 2020; Larsen et al., 2020; Wołyniec et al., 2020; Benedetti et al., 2021; Gaggini et al., 2021; Skinner et al., 2021; Sliwicka et al., 2021). Of this total, only the studies of Reihmane et al. (2013) and Costello et al. (2020) (3%) did not show increased concentration of IL-6 after half-marathon running.

### Interleukin 1β

Nineteen studies were found that evaluated the IL-1β (Drenth et al., 1995; Ostrowski et al., 1998, 1999; Neidhart et al., 2000; Suzuki et al., 2000, 2003; Nieman et al., 2001; Uchakin et al., 2003; Ng et al., 2008; Gill et al., 2015a,b; Luna Junior et al., 2016; Santos et al., 2016; Clifford et al., 2017; Passos et al., 2019; Sierra et al., 2019a; Batatinha et al., 2020; dos Santos et al., 2020; Skinner et al., 2021). In these studies, 20 runs were evaluated [half-marathon (*n* = 1), ultra-marathon (*n* = 4), marathon (*n* = 14) and 40 km running (*n* = 1)], of this total, (100%) half-marathon (Ng et al., 2008) and 40 km (Skinner et al., 2021), (50%) marathon (Neidhart et al., 2000; Suzuki et al., 2000; Uchakin et al., 2003; Luna Junior et al., 2016; Santos et al., 2016; Batatinha et al., 2020; dos Santos et al., 2020) e (25%) ultra-marathon (Drenth et al., 1995), did not show increased concentration of IL-1β. When the behavior of IL-1β between two tests was analyzed (40 km vs 171 km) in the same study, IL-1β was increased only in the ultra-marathon running (Skinner et al., 2021).

### Interleukin-1 Receptor Antagonist

Twenty-three studies were found that evaluated interleukin-1 receptor antagonist (IL-1ra), in all run volumes evaluated (Drenth et al., 1995; Castell et al., 1996; Ostrowski et al., 1998, 1999, 2000; Neidhart et al., 2000; Suzuki et al., 2000; Toft et al., 2000; Nieman et al., 2001, 2002, 2003, 2005, 2006, 2007; Peters et al., 2001; Pistilli et al., 2002; Ng et al., 2008; Cox et al., 2010; Díaz-Castro et al., 2012; Abbasi et al., 2013; Santos et al., 2013a; Gill et al., 2015b; Clifford et al., 2017). Of this total, only the study of Clifford et al. (2017) (3%) showed no elevation in the concentration of IL-1ra after marathon running.

### Interleukin 2

Eight studies were found that evaluated IL-2, in two runs studied, being six marathons (Castell et al., 1996; Suzuki et al., 2000; Santos et al., 2013b; Clifford et al., 2017; Batatinha et al., 2020; Tavares-Silva et al., 2021) and two ultra-marathon (Nieman et al., 2002; Skinner et al., 2021). Of this total, the studies of Castell et al. (1996), Suzuki et al. (2000) and Clifford et al. (2017) showed no decrease in IL-2 concentration and the studies by Nieman et al. (2002) and Skinner et al. (2021) demonstrated an increase in IL-2 concentration.

### Interleukin 4

Seven studies were found that evaluated IL-4 (Suzuki et al., 2000; Luna Junior et al., 2016; Clifford et al., 2017; Batatinha et al., 2020; dos Santos et al., 2020; Skinner et al., 2021; Tavares-Silva et al., 2021). Of this total, only the study of Skinner et al. (2021) found an increase in IL-4 after a 171 km run.

### Interleukin 8

Twenty-seven studies were found that evaluated IL-8, in the distances between half-marathon and ultra-marathon running (≤ 208 km) (Niess et al., 1999; Fehrenbach et al., 2000; Suzuki et al., 2000, 2003; Nieman et al., 2001, 2002, 2003, 2005, 2006, 2007; Pistilli et al., 2002; Cox et al., 2010; Abbasi et al., 2013; Shanely et al., 2014; Bachi et al., 2015; Gill et al., 2015a; Luna Junior et al., 2016; Niemelä et al., 2016; Santos et al., 2016; Clifford et al., 2017; Passos et al., 2019; Pugh et al., 2019; Sierra et al., 2019a,b; Batatinha et al., 2020; dos Santos et al., 2020; Skinner et al., 2021). Of this total, only the studies of Bachi et al. (2015), Luna Junior et al. (2016) and Sierra et al. (2019a) (11%) showed no increase in IL-8 concentration after marathon running.

### Interleukin 10

Thirty-three studies were found that evaluated IL-10, about half-marathon distances (Ng et al., 2008; Cox et al., 2010; Abbasi et al., 2013; Niemelä et al., 2016), marathon (Suzuki et al., 2000, 2003; Nieman et al., 2001; Pistilli et al., 2002; Scherr et al., 2011; Nickel et al., 2012; Santos et al., 2013b, 2016; Vaisberg et al., 2013; Shanely et al., 2014; Bachi et al., 2015; Luna Junior et al., 2016; Niemelä et al., 2016; Clifford et al., 2017; Passos et al., 2019; Pugh et al., 2019; Sierra et al., 2019b; Batatinha et al., 2020; dos Santos et al., 2020; Larsen et al., 2020; Tavares-Silva et al., 2021) and ultra-marathon (Peters et al., 2001; Nieman et al., 2002, 2003, 2005, 2006, 2007; Shin and Lee, 2013; Gill et al., 2015a,b). Of this total, one study did not showed an increase in the concentration of IL-10 after marathon running (Luna Junior et al., 2016). Furthermore, the study by Santos et al. (2013b) showed a reduction after marathon running.

### Tumor Necrosis Factor-α

Forty-three studies were found that evaluated TNF-α. One study show a reduction in TNF-α concentration, after marathon running (Santos et al., 2013b). Nineteen investigations, showed no differences in TNF-α immediately after half-marathon, marathon and ultra-marathon running (Drenth et al., 1995; Castell et al., 1996; Fehrenbach et al., 2000; Bonsignore et al., 2002; Suzuki et al., 2003; Nieman et al., 2006; Kim et al., 2007; Ng et al., 2008; Nickel et al., 2012; Abbasi et al., 2013; Reihmane et al., 2013; Santos et al., 2013a, 2016; Luna Junior et al., 2016; Passos et al., 2019; Sierra et al., 2019b; Batatinha et al., 2020; Costello et al., 2020; Gaggini et al., 2021). Regarding the studies that showed an increase in TNF-α levels, such results were observed after half-marathon (Bonsignore et al., 2002; Zimmer et al., 2016), marathon (Ostrowski et al., 1998, 1999; Neidhart et al., 2000; Toft et al., 2000; Nieman et al., 2001; Starkie et al., 2001; Uchakin et al., 2003; Scherr et al., 2011; Vaisberg et al., 2012; Bernecker et al., 2013; Wilhelm et al., 2014; Clifford et al., 2017; dos Santos et al., 2020; Larsen et al., 2020; Sliwicka et al., 2021; Tavares-Silva et al., 2021), ultra-marathon with variability of distances between 50 and 230 km (Nieman et al., 2007; Díaz-Castro et al., 2012; Chiu et al., 2013; Gill et al., 2015a,b; Skinner et al., 2021) and in 40 km run (Skinner et al., 2021).

### Interferon Gamma

The Interferon gamma (IFN-y) was evaluated in nine studies (Suzuki et al., 2000; Nieman et al., 2002; Henson et al., 2004; Abbasi et al., 2013; Gill et al., 2015a,b; Clifford et al., 2017; Batatinha et al., 2020; Skinner et al., 2021). There was no change in IFN-γ concentration in six studies (Suzuki et al., 2000; Abbasi et al., 2013; Gill et al., 2015b; Clifford et al., 2017; Batatinha et al., 2020; Skinner et al., 2021). In two studies, a decrease in IFN-γ concentration was found after running (Nieman et al., 2002; Henson et al., 2004), 80 km and marathon, respectively.

### Leptin, Resistin, Adiponectin, and Visfatin

Five studies were found that evaluated the Leptin (Zaccaria et al., 2002; Bernecker et al., 2013; Roupas et al., 2013; Vuolteenaho et al., 2014; Sansoni et al., 2017). In the half-marathon and marathon running there were no significant changes (Zaccaria et al., 2002; Bernecker et al., 2013; Vuolteenaho et al., 2014). In the ultra-marathon running, Leptin levels reduced after the running (Zaccaria et al., 2002; Roupas et al., 2013; Sansoni et al., 2017). The Resistin was evaluated in four studies showing increased concentration after running (Roupas et al., 2013; Vuolteenaho et al., 2014; Sansoni et al., 2017; Czajkowska et al., 2020). Two studies were found that evaluated the concentration of adiponectin (Roupas et al., 2013; Vuolteenaho et al., 2014). There was an increase in adiponectin concentration after the marathon (Vuolteenaho et al., 2014). The Visfatin was evaluated in two studies (Roupas et al., 2013; Sansoni et al., 2017). Visfatin concentration increased only in the study that presented the lowest running volume 65km (Sansoni et al., 2017).

### IL-12, IL-12p40, IL-12p70, IL-23, IL-33, IL-15, IL-7, IL-17a, IL-3, and IL-5

The IL-12 was evaluated in two studies: (Santos et al., 2016; Skinner et al., 2021). There was no significant difference after the run, regardless of the distance covered. Two studies evaluated the IL-12p40: (Abbasi et al., 2013; Sierra et al., 2019a). The concentration of IL-12p40 was reduced after the marathon (Sierra et al., 2019a). The IL-12p70 was also evaluated in three studies (Abbasi et al., 2013; Passos et al., 2019; Sierra et al., 2019b). There was no change in IL-12p70, regardless of running volume. Only one study evaluated the IL-23 e IL-33: (Sierra et al., 2019a), both cytokines reduced concentration after marathon (Sierra et al., 2019a). Only one study evaluated IL-15 and there was no change (Batatinha et al., 2020). IL-7 and IL-17a were evaluated in only one study (Skinner et al., 2021). Increases in the concentration of both cytokines were found after 170 km of running. Only one study evaluated IL-3 and IL-5 and there was no change (Skinner et al., 2021).

### VEGF-A, Fractalkine, sTNFRII, sIL-6R, TGF-β, G-CSF, GM-CSF, MCP-1, MIP-1β, HSP72, HSP27, and HSP70

Only one study evaluated VEGF-A, finding no differences after the half-marathon, on the other hand fractalkine, showed an increase in its concentration after the half-marathon (Gaggini et al., 2021). The sTNFRII and sIL-6R were evaluated in a study (Neidhart et al., 2000), there was no change after marathon. Three studies evaluated the TGF-β (Toft et al., 2000; Abbasi et al., 2013; Niemelä et al., 2016) and no significant differences were found after running. The G-CSF was evaluated in seven studies (Suzuki et al., 2000, 2003; Bonsignore et al., 2002; Nieman et al., 2005, 2006, 2007; Shanely et al., 2014). G-CSF concentration increased after running in all studies, regardless of running distance. Eight studies evaluated MCP-1 (Suzuki et al., 2003; Nieman et al., 2005, 2006, 2007; Abbasi et al., 2013; Shanely et al., 2014; Clifford et al., 2017; Skinner et al., 2021) and only in one study there was no change in the concentration of MCP-1 (Clifford et al., 2017). Four studies evaluated the MIP-1β (Nieman et al., 2005, 2006, 2007; Skinner et al., 2021) and showed concentration increase. The GM-CSF was evaluated in one study (Skinner et al., 2021) and did not showed alterations. Two studies evaluated HSP72, there was an increase in concentration after running in both studies (Shanely et al., 2014; Yargic et al., 2019). The HSP27 e HSP70 were evaluated in only one study (Bekos et al., 2019). Both showed an increase after the half-marathon and marathon.

### Meta-Analysis

**Figure 4:**
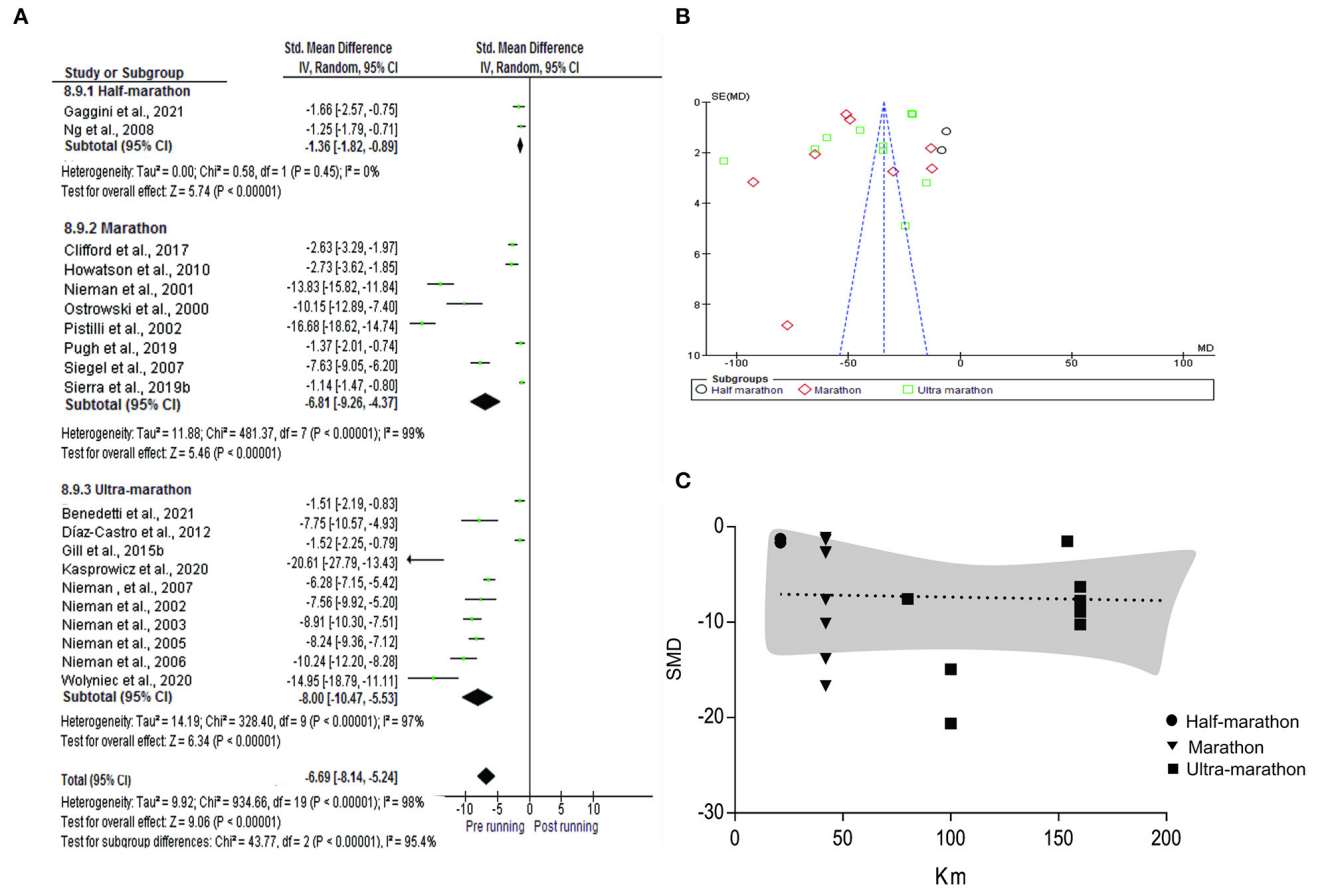
Meta-analysis of half-marathon, marathon, and ultra-marathon. **(A)** Forest plot, **(B)** Funnel plot, and **(C)** meta-regression analyses.

For the meta-analysis, were included the individual and summarized effects of the studies that analyzed the IL-6 (*n* = 20), IL-1ra (*n* = 10), IL-Iβ (*n* = 7), IL-2 (*n* = 5), IL-4 (*n* = 3), IL-8(*n* = 13), IL-10 (*n* = 12), TNF-α (*n* = 11) and INF-y (*n* = 6). In the analysis of subgroups performed with IL-6 in consideration of half-marathon (*n* = 2), marathon (*n* = 8) and ultra-marathon (*n* = 10), in the pre vs post-running comparison, an increase was observed in all the distances (SMD −6.69; IC 95%: −8.14, −5.24; *p* < 0.0001) highlighted a regular rise to running distance: half-marathon (SMD −1.36; IC 95%: −1.82, −0.89, Ch^2^:0.58; tau^2^:0.00; *p* < 0.0001), marathon (SMD −6.81; IC 95%: −9.26, −4.37; Ch^2^:481.37 tau^2^:11.88; *p* < 0.0001) and ultra-marathon (SMD−8.00 IC 95%: −10.47,−5.53; Ch^2^:328.40; tau^2^:14.19; *p* < 0.0001). Meta-regression analysis showed no significant regressions on half marathon, marathon and ultra-marathon (p= 0.864) (Figure 4).

**Figure 5:**
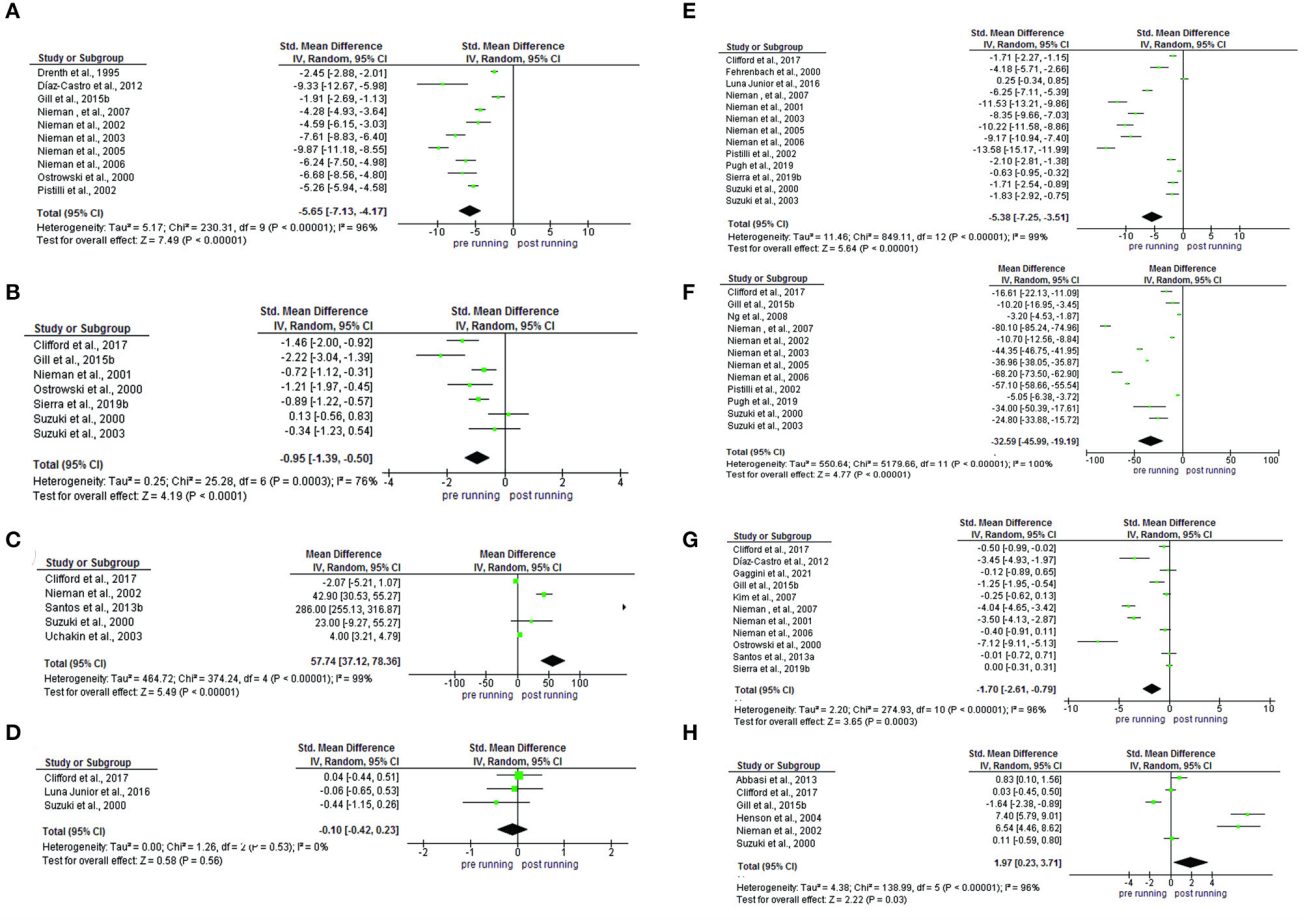
Comparison and effect among cytokines **(A)** IL-1ra, **(B)** IL-1β, **(C)** IL-2, **(D)** IL-4, IL**(E)**−8, **(F)** IL-10, **(G)** TNF-α, and **(H)** INF-y.

An increase in the concentration of IL-1ra (SMD−5.65; IC 95%:−7.13,-4.17; *p* < 0.0001), IL-1ra (SMD −0.95; IC 95%: −1.39,-0.50; *p* < 0.0001), IL-8 (SMD −5.38; IC 95%: −7.25,-3.51; *p* < 0.0001), IL-10 (SMD −32.59; IC 95%: −45.99,-19.19; *p* < 0.0001) and TNF-α (SMD −0.83; IC 95%: −1.00,-0.67; *p* < 0.0001). A reduction in the concentration of IL-2 (SMD 57.74; IC 95%: 37.12, 78.36; *p* < 0.0001) and INF-y (SMD 1.97; IC 95%: 0.23,3.71; *p* < 0.03) and there was no change in the IL-4 (SMD −0.10; IC 95%: −0.42,0.23; *p* < 0.56). Evidence of heterogeneity and inconsistency was found for IL-1ra (Ch^2^ = 230.31; Tau^2^ =5.17; I^2^ = 96%), IL-1ra (Ch^2^ = 25.28; Tau^2^ = 0.25; I^2^ = 76%), IL-2 (Ch^2^ = 374.24; Tau^2^ = 464.72; I^2^ = 99%), IL-8 (Ch^2^ =849.11; Tau^2^ = 11.46; I^2^ = 99%), IL-10 (Ch^2^ =550.64; Tau^2^ = 5,179.66; I^2^ = 96%), TNF-α (Ch^2^ =274.93; Tau^2^ = 2.20; I^2^ = 96%) and INF-y (Ch^2^ =138.99; Tau^2^ = 4.38; I^2^ = 96%) (Figure 5).

## Discussion

In the current report, we describe changes in impacts of running volume on the concentration of cytokines in half-marathon, marathon and ultra-marathon. Was verified increase in inflammation status immediately after completing the running. The meta-analysis showed that the running volume variability modulate in the maintenance of homeostasis and cell signaling. On the other hand, the increase in volume does not proportionally increase the concentration of IL-6, IL-1ra, IL-1β, IL-8, IL-10 and TNF-α after the running. In contrast, few studies evaluated adipokines. Prolonged aerobic exercise exerts a huge impact on metabolism and energy balance, is an exhausting effort for the whole organism and leads to a pro-inflammatory profile.

Cytokines are important mediators regularizing the immune response, and their enhancement may yield valuable information pertinent to questions like transient post-running immunosuppression, beneficial anti-inflammatory. Although a high increase in cytokine concentration was expected to occur after long-distance running, a key point was if an increase in concentration proportional to running volume would occur. This fact was not confirmed when analyzing the studies that evaluated the concentration of IL-6 by subgroups: half-marathon, marathon and ultra-marathon (Figure 3). Regardless of if running over 42 km are more complex, results show that to achieve homeostatic equilibrium, the integrated action of the neuroendocrine and immune systems is necessary (Bachi et al., 2015). The higher serum levels of growth hormone in athletes at rest and the higher production of cytokines without previous stimulus suggests that long distance runners present mechanisms that may be associated with preparing the body to perform prolonged strenuous exercise. Therefore, the inherent vulnerability to exercise induced inflammatory alterations is passible adaptation mechanisms to training (Scherr et al., 2011).

Regardless of whether long-distance running athletes are more prepared, the studies that evaluated the recovery time of IL-6 showed after 21km, the less time required to return to baseline conditions. So, several hypotheses have been raised to explain that recovery seems to be more associated with running volume including intensity of exercise or training status (Scherr et al., 2011; Gaggini et al., 2021). However, the results are still controversial. If on the one hand, in the quantification of the concentration of IL-6 after a half-marathon, it was only verified an increase immediately after the running, occurs a return to baseline conditions after the running (Gaggini et al., 2021) or also even 30 minutes (Abbasi et al., 2013). And that pattern was different after marathon, kinetics of serum IL-6 concentration prolonged elevation for at least 24 h (Scherr et al., 2011). Furthermore, the studies that evaluated 100 km (Chiu et al., 2013) and 50 km (Mastaloudis et al., 2004) showed baseline conditions in 24 h.

In accordance with the reports of studies, was verified that levels of six cytokines, IL-6, IL-1ra, IL-1β, IL-8, IL-10 and TNF-α rose strongly in response to race competition. Unlike IL-2 and INF-y, whom decreased. Post running levels of IL-4, remained near pre running or at no detectable levels. This outcome corroborates with classical studies (Nieman et al., 2001). Reported long- running induced muscle cell metabolic activity and damage appear to be important triggers of macrophage and neutrophil migration and cytokine release (Terra et al., 2012). The low post running levels found in studies as IL-4 may be due to the strong inhibitory effects of IL-10, IL-1ra and IL-6 which together help prevent an overly active systemic inflammation (Suzuki et al., 2000). Several explain about the decrease in the IL-2 and INF-y reported increased lymphocyte proliferation (Santos et al., 2013b). Leukocyte function is modulated by different pathways such as proliferation control, cytokine and anti-inflammatory mediator, generation, adhesion molecule expression, and cell death (Akhtar Khan, 2010).

Physical activity increases myokine levels. The IL-6 was observed on a large scale in 62 studies. IL-6 plays a positive role in glucose metabolism (Glund and Krook, 2007; Shoghi et al., 2008; Pedersen, 2011), increasing its uptake by myocytes (Carey et al., 2006), and elevating insulin-stimulated glycogen synthesis in skeletal muscle (Weigert et al., 2005). Intramuscular concentration of IL-6 mRNA (Keller et al., 2001) and protein release (Steensberg et al., 2001) are exacerbated when intramuscular glycogen is compromised, suggesting that IL-6 functions as an energy sensor (Pedersen and Febbraio, 2008). The level of IL-6 was shown to increase exponentially proportional to the exercise duration and the amount of muscle mass involved in the exercise (Pedersen, 2016). Running, which involves several large muscle groups, is the mode of exercise where most marked increases in plasma IL-6 have been observed (Pedersen and Febbraio, 2008). Exercise duration is the most important factor in determining post-exercise plasma IL-6 amplitude (Reihmane et al., 2013). In this perspective, plasma IL-6 can increase up to 40 times after a marathon (Reihmane et al., 2013; Santos et al., 2016; Larsen et al., 2020; Skinner et al., 2021), with the elevation being equivalent to that observed in negative health status (Skinner et al., 2021).

The Inflammatory response is associated with the pro-inflammatory cytokine storm (Smith et al., 2020). This process is due in part to the activation of M1 type macrophages, which have characteristic proinflammatory such as IL-1β (Bent et al., 2018). This important cytokine is one of the mediators of inflammation and is involved in several cellular activities, including cell proliferation, differentiation, and apoptosis (Conti et al., 2002; Bent et al., 2018). The present review identified variability in the effects of running volume on IL-1β levels. The majority demonstrated significant increase in long-distance runners. Thus, the increased plasma concentrations of IL-1β after the Prolonged aerobic exercise could reflect exercise-mediated inflammasome NLRP3 pathway activation during long-running caused by the extreme effort. Oxidized hemoglobin, a hemolysis related product, has been identified as a potent trigger of NLRP3 activation and IL-1β production (Skinner et al., 2021).

Interleukins 2, 4, and 8 actively participate in the structure of the immune system, in the maturation of T lymphocytes, activation of macrophages *via* alternative pathways and migration of neutrophils, respectively. Few studies have evaluated the responses caused by running volume in IL-2 and 4. Only two studies identified a reduction in IL-2 and INF-y levels in marathon runners, studies showed that low levels of this protein are associated with impairment of immunological memory and character pathologies inflammatory including rheumatoid arthritis (Arenas-Ramirez et al., 2015; Wu et al., 2020). In a previous study, was reported a significant reduction of IL-2 and INF-y production in runners at the completion of an 80 km ultra-marathon (Nieman et al., 2002). In addition, other exhaustive aerobic exercise depression of IL-2 and INF-y 1 h after the event (Weinstock et al., 1997; Nieman et al., 2005) and after and 1.5 h after completion of the marathon race (Henson et al., 2004). These findings are consistent with our data showing a significant decline in both induced production of INF-y (unadjusted and adjusted per T-cell) and IL-2. Additionally, in IL-4 levels, no differences were found in runners. Inhibitory cytokines, namely IL-4 have been shown to upregulate IL-1ra production and downregulate IL-1β and TNF-α (Suzuki et al., 2000). Cortisol concentration also increased after the race, and it has been shown that it can also inhibit the production of several cytokines (Nieman et al., 2001).

Most of the thirteen studies used in our systematic review demonstrated high levels of IL-8 in the blood of half-marathon, marathon, and 160 km runners. Studies demonstrate the effective participation of physical activity and physical exercise, especially the aerobic emphasis on the modulation of this myokine (Barbalho et al., 2020). IL-8 is a classical proinflammatory cytokine, it was originally identified as a chemoattractant factor for neutrophils (dos Santos et al., 2020). Beyond its proinflammatory role, IL-8 also presents a prominent angiogenic function and, as mentioned, is considered a myokine (dos Santos et al., 2020). According to the literature, the increased IL-8 levels in response to muscle damage induced by a physical exercise session are mainly associated with the regulation of muscle angiogenesis by its binding to the CXC receptor 2 (CXCR2) expressed in microvascular endothelial cells in order to improve muscle regeneration (Frydelund-Larsen et al., 2007).

The IL-10 and TNF-α carry out antagonistic immune responses. Biologically, IL-10 works by deactivating macrophages to produce inhibitory effects on Natural Killer (NK) cells and T lymphocytes, moreover, to playing a fundamental anti-inflammatory role (Saraiva and O'Garra, 2010). Our data reveal that different running volumes significantly elevated blood and urine IL-10 levels. Pedersen (2017) demonstrated that physical exercise can potentiate IL-10 activity, reducing inflammation that is part of the synthesis of several cardiometabolic pathologies, including type 2 diabetes mellitus and cardiovascular diseases. This points to the possible preventive character of this immunological marker in these conditions (Pedersen, 2017).

On the other hand, TNF-α is identified as one of the considerable cytokines for inflammation in different organisms. Furthermore, it is linked to the emergence and development of different types of cancers (Balkwill, 2009). In the data used in this review, we found a variety of responses within the production of this protein after different running volumes. Furthermore, it is known that acute physical exercises that require high demands of metabolic effort, including ultra-marathon, can promote an increase in this cytokine (Uchida et al., 2014). Results indicate that during a marathon run, the pro-inflammatory markers TNF-α are not produced by blood mononuclear cells on the mRNA level to a clinically relevant extent during a marathon running (Bernecker et al., 2013). There is, however, a significant increase in TNF-α in the plasma, suggesting a local production or release from the stressed skeletal muscle tissues of these cytokines. More studies are needed in high running volumes for better understand (Uchida et al., 2014).

Were also observed changes caused by high running volume in leptin, resistin, adiponectin and visfatin levels. Leptin participates in the regulation of food intake, energy balance and reproductive system (Zhang and Chua, 2017). Furthermore, its deficiency in its production is related to the genesis of obesity. Only two studies looked at the possible effects of running volume on the production of this hormone in the blood. Where only, Zaccaria et al. (2002) observed a significant reduction in leptin after 100 km of running. Several studies with different experimental models demonstrate that aerobic exercise can bring changes in leptin levels, increasing its sensitivity and acting preventively on obesity (Yetgin et al., 2018; Fernandes et al., 2020).

In contrast, high levels of resistin were found in the blood of marathon runners and 100 km away. This protein is secreted by adipose tissue, immune and epithelial cells in mammals, it has the function of blocking the action of leptin, reducing satiety (Acquarone et al., 2019). Moreover, clinical studies have shown that the increase in resistin after physical exercise is associated with a reduction in fatigue, playing a fundamental role in the reduction of musculoskeletal and joint inflammatory events, common in pathologies such as fibromyalgia, obesity, and high demands for effort physical and running volumes (Bjersing et al., 2013).

Adiponectin's are proteins produced mainly because they play a fundamental role in glycemic and free fatty acid control. In our data, an increase in adiponectin levels in marathon runners was observed only in one study. However, the role of physical exercise in different modalities and protocols in the increase of adiponectin's is well established (Li et al., 2019). Finally, visfatin, it is produced by visceral adipose tissue with insulinomimetic activities with systemic and local action. Within the studies that evaluated this biomarker, no significant differences were observed. However, its role associated with physical exercise needs to be elucidated (Jamurtas et al., 2015).

In addition, we also consider other limitations that have been little explored in the research, mainly related to the analysis between sex, ultra-marathon distance and follow-up of 24, 48, and 72 h. Only 1 study discriminated the results differentiating by sex. Although 31% of studies evaluated 24-h follow-up, only 3% was evaluated in ultra-marathon running, of these, 48 and 72-h follow-up was evaluated in 2 and 1 studies, respectively. There was great variability of distance between the ultra-marathon running (51–308 km) generating high amplitude (6:00 to 62:20 h) between the times of conclusion of the races. All these facts, added to the heterogeneity, made it difficult to carry out a quantitative analysis, suggesting the expansion of new studies contemplating these objectives, as well as, differentiating single and multi-day ultra-marathons.

**Figure 6:**
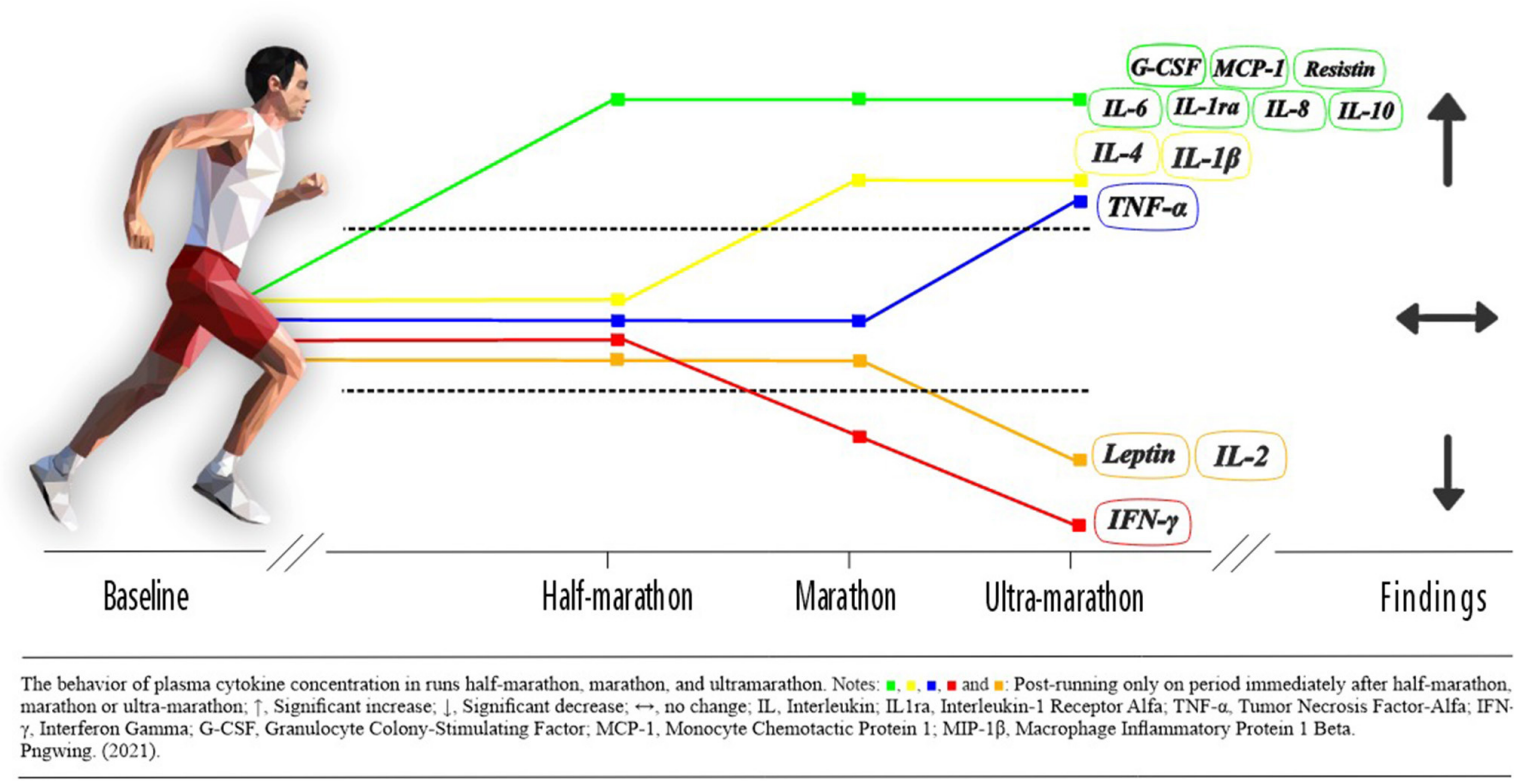
Changes in cytokines concentration following long-distance running (Pngwing, 2021).

In conclusion, the trans-signaling of cytokines results in inflammation and is therefore linked to high-grade inflammatory. Long-distance running promote an increase in the concentration of IL-6, IL-1ra, IL-1β, IL-8, IL-10 and TNF-α and a decrease in the concentration of IL-2 (Figure 6). The effects of an acute bout of prolonged aerobic exercise will protect against chronic systemic inflammation. The time to return to baseline values showed a substantial and dose-dependent relationship with run volume. The concentration of IL-6 was robustly studied in long-distance running present in more than 80% of the selected articles and the marathon running was the most explored. Further studies with adipokines are recommended mainly in ultra-marathons as well, and further investigations related to runner level, age, sex, follow-up, multi-stage ultra-marathons also suggested.

In practical considerations, the ability of a runner to minimize the effects of an acute bout of prolonged aerobic exercise will against chronic systemic inflammation may be identified as one of the determinant factors of performance. The athletes attention must be taken in age regulations, nutrition, training status and race-specific factors (elevation change, such as distance, level of medical assistance, ambient temperatures and type of provisions provided by the race organizers), being able decrease induced physical and physiological distresses and speeding up recovery and rehabilitation from injuries. Long-distance running may have different physiological requirements for ultra-marathon, marathon, and half-marathon. Consequently the strategies of runners provide a challenge for inflammatory process, and there is a general interest in interpret its alterations.

## Data Availability Statement

The raw data supporting the conclusions of this article will be made available by the authors, without undue reservation.

## Author Contributions

MA, DS, and RS: conceptualization. MA, DS, EP, and DO: methodology. RS, MS, DP, MA, and DS: writing–original draft. FA, DS, and LV-S: formal analysis. RS, MA, DS, EP, DO, MS, and FA: writing-review and editing. All authors contributed to the final version of the manuscript.

## Funding

We would like to thank the Coordination for the Improvement of Higher Education Personnel (CAPES, Finance Code 001 and CAPES/PROCAD-2013).

## Conflict of Interest

The authors declare that the research was conducted in the absence of any commercial or financial relationships that could be construed as a potential conflict of interest.

## Publisher's Note

All claims expressed in this article are solely those of the authors and do not necessarily represent those of their affiliated organizations, or those of the publisher, the editors and the reviewers. Any product that may be evaluated in this article, or claim that may be made by its manufacturer, is not guaranteed or endorsed by the publisher.
